# ZNF384: A Potential Therapeutic Target for Psoriasis and Alzheimer’s Disease Through Inflammation and Metabolism

**DOI:** 10.3389/fimmu.2022.892368

**Published:** 2022-05-20

**Authors:** Shougang Liu, Xiuqing Yuan, Hang Su, Fanghua Liu, Zhe Zhuang, Yongfeng Chen

**Affiliations:** Dermatology Hospital of Southern Medical University, Guangzhou, China

**Keywords:** psoriasis, Alzheimer’s disease, inflammation, metabolic disorders, transcription factors

## Abstract

**Background:**

Psoriasis is an immune-related skin disease notable for its chronic inflammation of the entire system. Alzheimer’s disease (AD) is more prevalent in psoriasis than in the general population. Immune-mediated pathophysiologic processes may link these two diseases, but the mechanism is still unclear. This article aimed to explore potential molecular mechanisms in psoriasis and AD.

**Methods:**

Gene expression profiling data of psoriasis and AD were acquired in the Gene Expression Omnibus (GEO) database. Gene Set Enrichment Analysis (GSEA) and single-sample GSEA (ssGSEA) were first applied in two datasets. Differentially expressed genes (DEGs) of two diseases were identified, and common DEGs were selected. Kyoto Encyclopedia of Genes and Genomes (KEGG) pathway enrichment analysis was performed to explore common biological pathways. Signature transcription factors (STFs) were identified and their diagnostic values was calculated by receiver operating characteristic (ROC) curve analysis in the exploration cohort and verified in the validation cohort. The expression levels of STFs were further investigated in the validation cohort and the GTEx Portal Database. Additionally, four kinds of interaction analysis were performed: correlation analysis among STFs, gene-gene, chemical-protein, and protein-ligand interaction analyses. In the end, we predicted the transcription factor that potentially regulates STFs.

**Results:**

Biosynthesis and metabolic pathways were enriched in GSEA analysis. In ssGSEA analysis, most immunoreaction gene lists exhibited differential enrichment in psoriasis cases, whereas three receptor-related gene lists did in AD. The KEGG analysis of common DEGs redetermined inflammatory and metabolic pathways essential in both diseases. 5 STFs (PPARG, ZFPM2, ZNF415, HLX, and ANHX) were screened from common DEGs. The ROC analysis indicated that all STFs have diagnostic values in two diseases, especially ZFPM2. The correlation analysis, gene-gene, chemical-protein, and protein-ligand interaction analyses suggested that STFs interplay and involve inflammation and aberrant metabolism. Eventually, ZNF384 was the predicted transcription factor regulating PPARG, ZNF415, HLX, and ANHX.

**Conclusions:**

The STFs (PPARG, ZFPM2, ZNF415, HLX, and ANHX) may increase the morbidity rate of AD in psoriasis by initiating a positive feedback loop of excessive inflammation and metabolic disorders. ZNF384 is a potential therapeutic target for psoriasis and AD by regulating PPARG, ZNF415, HLX, and ANHX.

## Introduction

Psoriasis is a chronic, immune-mediated skin disease characterized by plaques and scales (1). It affects about 2% of the world’s population and causes a tremendous economic burden. Plaque psoriasis is the most common form of psoriasis that accounts for 90% of cases (2). Innate immune cells such as dendritic cells, NK cells, and macrophage cells trigger and amplify topical inflammation; the adaptive immune cells like TH1and Th17 cells serve as critical components involving IL-17 and TNF-α signaling pathways (3). Biologics targeting IL-17A and TNF-α have demonstrated greater effectiveness than traditional strategies in treating psoriasis for decades. Since biologics became the first-line protocol in treating moderate to severe psoriasis, a new era of psoriasis treatment has begun (4). However, psoriasis chronic inflammatory states are not confined to the skin but affect the whole body, leading to multiple comorbidities, such as vascular diseases, metabolic syndromes, and depression. Some guidelines give recommendations for managing psoriasis comorbidities, which significantly facilitates the life quality of patients (5). However, adverse effects of hyperinflammatory states may not be limited to these acknowledged comorbidities. Therefore, identifying related diseases and exploring the common mechanisms will do great for psoriasis management.

Alzheimer’s disease (AD) is a neurodegenerative disease affecting more than 50 million people worldwide. It is the most common cause of dementia that finally robs people’s ability to live independently. Despite decades of research, the pathogenic mechanism is still unclear, and there exists no prescription to slow the progression of AD, let alone a curable therapy (6). The most classical hypothesis of pathogenesis is the accumulation of beta-amyloid peptides (Aβ) and hyperphosphorylated tau in the brain. The problem with this hypothesis is that it allows AD to be treated based on irreversible stages rather than one that can be cured. Finding earlier-stage targets of AD would be desirable so that action could be taken before irreversible damage occurs (7). Interestingly, in psoriasis cases, the morbidity of AD was statistically higher than the general population (8, 9). This phenomenon indicates that common signature genes and signaling pathways may exist and contribute to both diseases. Although some scholars supposed chronic inflammatory conditions might play a role in psoriasis and AD, the molecular mechanism is still not well understood (8). Furthermore, transcription factors regulate gene sets functioned in biological processes, whose aberrant expressions are considered the trigger of many diseases (10). Psoriasis and AD are no exceptions. In psoriasis, Nuclear factor kappa B (NF-κB), and aryl hydrocarbon receptor (AhR) are proved to be the crucial transcription factors for regulating inflammatory pathways (11, 12). While in AD, the repressor element 1-silencing transcription/neuron-restrictive silencer factor (REST/NRSF), signal transducer and activator of transcription 3 (STAT3), Nuclear factor erythroid 2 related factor 2 (NRF2) are demonstrated to be significant in the pathogenesis of AD, mainly through regulating gene networks that participate in apoptosis, autophagy, and inflammatory responses (13–15). Notably, targeting these transcription factors has been proved beneficial in psoriasis and AD (16, 17). Therefore, the common transcription factors involved in psoriasis and AD may not only help us understand the potential links between these two diseases but provide attractive therapeutic targets for them. Zinc finger protein 384 (ZNF 384) encodes C2H2-type zinc finger proteins and functions as a transcription factor contributing to extracellular matrix remodeling (18, 19). Anomalous expression of ZNF384 is associated with acute lymphoblastic leukemia (ALL), high-grade gliomas, hepatocellular carcinoma, and so on (20–22). Besides being a potential oncogene, studies also demonstrated that ZNF384 fusions might regulate immune-related pathways, the JAK-STAT signaling pathway, for example (23, 24). However, the research about ZNF384 in psoriasis and AD is vacant. Therefore, our research conducted a silico experiment to explore the common transcription factors and their upstream regulator in psoriasis and AD.

Using gene expression data downloaded from the Gene Expression Omnibus (GEO) (http://www.ncbi.nlm.nih.gov/geo/), we first conducted enrichment analyses on two diseases respectively. We identified immune and metabolism-related pathways essential in two diseases. Then, we screened common differentially expressed genes (DEGs) of psoriasis and AD. Kyoto Encyclopedia of Genes and Genomes (KEGG) pathway enrichment analysis of common DEGs reconfirmed inflammatory and metabolic pathways critical to the pathogenesis of both diseases. Five signature transcription factors (STFs) were identified, and the receiver operating characteristic (ROC) curve analysis was performed to calculate the diagnostic values of STFs in the exploration cohort and confirmed in the validation cohort. Gene expression levels of STFs were observed in the validation cohort and the GTEx Portal Database (https://www.gtexportal.org/) to validate their specificity in two diseases. We then conducted interaction analysis among STFs, relative genes, chemicals, and specific biologics. The results implicated that STFs might play pivotal roles in psoriasis and AD by aberrantly regulating inflammatory and metabolic processes. Finally, we found that ZNF384 might regulate STFs, thus influencing psoriasis and AD. To the best of our knowledge, our study might be the first research that explored the molecular mechanism between psoriasis and AD using a systems biology approach.

## Materials and Methods

### Data Acquisition

Data mining analyses for plaque psoriasis and AD were conducted based on the GEO database (http://www.ncbi.nlm.nih.gov/geo/). We selected the GEO series that must contain lesion and non-lesion skin of plaque psoriasis cases as the psoriasis cohort and contain hippocampus tissue of both AD and normal aged as the AD cohort. Both cohorts should consist of no less than 20 samples, with subgroups of each cohort of no less than 10 samples. In addition, GEO series deriving from the same sequencing platform would be preferable. Based on the criteria mentioned above, the GEO dataset numbered GSE41664, GSE5281, GSE53552, and GSE48350 were selected. Four datasets were derived from GPL570 [HG-U133_Plus_2] Affymetrix Human Genome U133 Plus 2.0 Array. As GSE5281 only contained 10 hippocampal tissues derived from AD and 13 from the normal aged, to control the sample size and composition of each disease, we performed simple random sampling in R version 4.0.2 to select 10 cases and 13 controls in the other three datasets, respectively, for our research. Then, we converted the probes to gene symbols based on the annotation document that came with the GEO platforms in Sangerbox (http://sangerbox.com/) for subsequent analyses.

### GSEA Analysis of KEGG Enrichment

Sangerbox tools (http://vip.sangerbox.com/) is a free online platform for data analyses and visualization. Gene Set Enrichment Analysis (GSEA) was used to identify enriched biological pathways associated with psoriasis and AD. The gene expression data of non-lesion skin of psoriasis patients and hippocampus tissue of normal aged people available for control groups were set as the baseline in each disease. Then the samples from the psoriatic lesion skin and AD’s hippocampus tissue were compared to the correspondent normal groups. The platform obtained the GSEA software (version 3.0) and downloaded the c2.cp.kegg.v7.4.symbols.gmt subset for GSEA analysis. P-value < 0.05 and FDR < 0.25 were considered statistically significant.

### SsGSEA Analysis of Immunoreaction Gene Lists

The immunoreaction gene lists, obtained from the ImmPort database (https://www.immport.org/), are composed of various innate and adaptive responses categories. Antigen process, natural killing cells are for the innate response; BCR, TCR signaling pathways for the adaptive response; cytokines, chemokines, TNF, interleukins, and corresponding receptors for immunoreaction stages. The use of immunoreaction gene lists could provide more accurate and detailed information on how immunity influences the pathogenesis of diseases (25). Single Sample GSEA (ssGSEA) was performed to calculate the enrichment scores of 16 specific immunoreaction gene lists in each sample of two diseases in R 4.0.2 “GSVA” package with parameter method as “ssgsea” in the Sangerbox platform. The differences of enrichment levels in each subgroup of both diseases were further visualized in boxplots, correspondingly. Immunoreaction categories with enrichment scores differed between case and control groups (p-value < 0.05) were considered statistically significant. The single sample enrichment scores were further scaled by Z-score and presented as heatmaps (R package “pheatmap”).

### Differentially Expressed Analysis

Differentially expressed analysis was performed in Sangerbox using the R package limma (version 3.40.6) to obtain DEGs in psoriasis and AD, respectively. DEGs of GSE41664 and GSE5281 were screened independently with the threshold of |log_2_FC| > 1 and p-value < 0.05.

### Identified Biological Pathways of Common DEGs

By applying Venn diagrams, overlapping DEGs of GSE41664 and GSE5281 identified the common DEGs. Kyoto Encyclopedia of Genes and Genomes (KEGG) pathway enrichment analysis of common DEGs was performed in the KOBAS-i database (http://kobas.cbi.pku.edu.cn/) to explore common biological pathways in psoriasis and AD. Pathways with a p-value < 0.05 were regarded as statistically significant and further visualized in a string diagram.

### Identification of STFs

The human transcription factors data was downloaded from a public database (http://humantfs.ccbr.utoronto.ca/). By overlapping transcription factors and common DEGs, STFs were identified for in-depth analyses.

### ROC Curve Analysis of STFs in the Exploration Cohort and the Validation Cohort

The diagnostic effectiveness of the STFs in GSE41664 and GSE5281 were predicted by the receiver operating characteristic (ROC) curve analysis using the OmicStudio tools (https://www.omicstudio.cn/). The area under the curve (AUC), sensitivity, and 1-specificity were calculated. Those genes with AUC values > 0.60 in both psoriasis and AD were considered with diagnostic values, and those with AUC values > 0.80 were considered with high diagnostic values. The AUC values in the exploration cohort and the validation cohort were compared to determine whether the prognostic values of STFs were reliable.

### Investigating Gene Expression Levels of STFs in the Validation Cohort and in the GTEx Portal Database

To validate the expression levels of STFs in psoriasis and AD, we selected gene expression profiling data of STFs in GSE53552 and GSE48350. Combined with the sample grouping information, we presented STFs expression levels in boxplots by applying Sangerbox tools. Gene expression levels differentiated (p-value < 0.05) between case and control groups were regarded as statistically significant. To further validate the tissue specificity of STFs in the normal skin and hippocampus, we put each STF in the GTEx Portal database (https://www.gtexportal.org/) to observe their genotype-tissue expression levels.

### Correlation Analysis of STFs

The Pearson correlation analysis was used to investigate interaction relationships among STFs. Pearson correlation analysis of STFs in GSE41664 and GSE5281 was operated by “psych,” “corrplot version 0.92”, and “PerformanceAnalytics” R packages. P-value < 0.05 and Pearson correlation coefficient (PCC) > 0. 85 were considered as correlated, while p-value < 0.05 and PCC > 0. 95 strongly correlated.

### Gene-Gene Interaction Analysis

The UCSC Genome Browser database (http://genome.ucsc.edu/cgi-bin/hgGeneGraph) is a viewer that provides access to genome annotations and genomics data visualization (26). The gene interactions tool was applied to explore validated or potential interactions and pathways based on curated databases and text mining. The gene networks were built with PPARG, ZFPM2, ZNF415, HLX, and ANHX as the center, respectively. Based on degree values in Cytoscape version 3.9.0, the top 25 genes interacting with each STF were selected and further visualized in a circle map.

### Chemical-Protein Interaction Analysis

The STITCH database (http://stitch.embl.de/) is a search tool that provides integrated information on genes at the expression level. It is convenient for researchers to get a holistic overview of what chemicals or proteins may interact with the specific protein involved (27). Hence, we used this online database to investigate the validated or potential relationships among chemicals, proteins, and each STF. The STFs-related interaction with a combined score > 0.80 was defined as having statistical significance and deserved further exploration.

### Protein-Ligand Interaction Analysis

Autodock is an application developed for predicting the interaction between small-molecule ligands and macromolecules receptors, mainly used in drug design, discovery, and virtual screening based on computational algorithms (28). To predict bound conformations and free energies of binding for IL-17A or TNF-α antagonist ligands to macromolecular proteins of STFs, analyses proceeded in three steps: first, 3D protein structures of STFs were obtained in the UniProt database (https://www.uniprot.org/) as the receptors; second, the 3D crystal structures of the IL-17A and TNF-α antagonists were acquired from the RCSB database (https://www.rcsb.org/) as the ligands; finally, Autodock version 4.2.6 and MGLTools-1.5.7 were conducted to predict the protein-ligand interactions, while Pymol version 4.6.0 was applied to remove the solvent of structures before molecular docking and visualize each docking pocket of STFs binding to small ligands with optimal affinity. Docking pockets with binding energy lower than -2 kcal/mol were regarded as ideal binding conformations.

### Prediction of Transcription Factors Regulating STFs

Exploring whether these STFs were regulated collectively by transcription factors upstream, we used the NCBI database (https://www.ncbi.nlm.nih.gov/), the Jaspar database (https://jaspar.genereg.net/), and the UCSC database (http://genome.ucsc.edu/) to predict upstream regulators. Based on the NCBI database, 2,000 bp upstream and 100 bp downstream of each STFs (PPARG, ZFPM2, ZNF415, HLX, ANHX) initiation site was selected as the promoter sequence. Subsequently, based on STFs promoter regions, JASPAR 2020 and UCSC databases were utilized to predict the transcription factors of each STF and their potential binding locations. Finally, a relative profile score threshold of 90% was set to screen potential binding sites in the JASPAR database.

## Results

### Information of Expression Profiling Data

Upon the criteria set for data mining, four GEO datasets were selected. there are GSE41664, GSE5281, GSE53552, GSE48350. On the one hand, GSE41664 and GSE53552 contain lesion and non-lesion skin biopsy tissue from plaque psoriasis, with GSE41664 derived from mild to moderate cases and GSE53552 from moderate to severe cases. On the other hand, GSE5281 and GSE48350 contain the hippocampus tissue from AD and the normal aged, both subgroups aged > 63 years. The information of the selected four datasets was summed up in **Table 1**, GSE number, platforms, samples, tissue type, etc. We paired GSE41664 and GSE5281 as the exploration cohort for further analyses and paired GSE53552 and GSE48350 as the validation cohort to verify diagnostic values and expression levels of STFs.

**Table 1:** Information of selected four datasets.

ID	GSE number	Platform	Samples	Tissue type	Disease	Group
1	GSE41664	GPL570	10 PP and 13 PN	skin	Psoriasis	Exploration cohort
2	GSE5281	GPL570	10 AD and 13 NOR	hippocampus	AD	Exploration cohort
3	GSE53552	GPL570	10 PP and 13 PN	skin	Psoriasis	Validation cohort
4	GSE48350	GPL570	10 AD and 13 NOR	hippocampus	AD	Validation cohort

PP, lesion skin tissue of plaque psoriasis; PN, non-lesion skin tissue of plaque psoriasis; AD, hippocampus tissue of AD; NOR, hippocampus tissue of the normal aged.

### Enriched KEGG Pathways in GSEA Analysis

**Figure 1:**
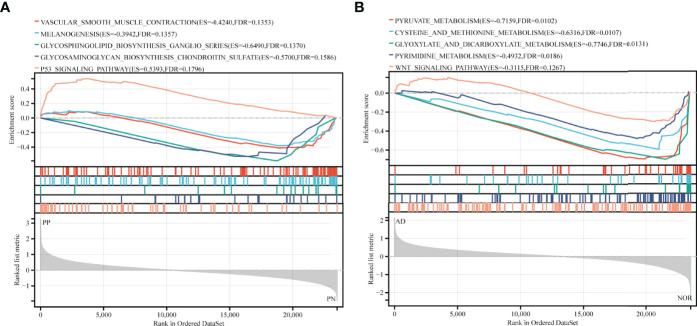
GSEA analysis in exploration cohort (GSE41664 for psoriasis and GSE5281 for AD). **(A, B)** GSEA plots for five KEGG pathways significantly enriched in GSE41664 and GSE5281. Screening criteria of selected KEGG pathways: biological functions of pathways, FDR value < 0.25, and p-value < 0.05. ES, Enrichment Score, FDR, False Discovery Rate.

31 KEGG pathways were enriched in GSE41664, while 45 KEGG pathways enriched in GSE5281. Pathways in both diseases mainly related to biosynthesis and metabolic processes (**Supplementary Table 1**). By combining the main biological function of pathway and its FDR value, and p-value, we selected 5 KEGG pathways ranked by FDR value: Vascular smooth muscle contraction (FDR = 0.135, p-value = 0.034), Melanogenesis (FDR = 0.136, p-value = 0.012), Glycosphingolipid biosynthesis ganglio-series (FDR = 0.137, p-value = 0.006), Glycosaminoglycan biosynthesis keratan sulfate (FDR = 0.159, p-value = 0.01), and P53 signaling pathway (FDR = 0.180, p-value = 0.006) in psoriasis. Pyruvate metabolism (FDR = 0.010, p-value < 0.001), Cysteine and methionine metabolism (FDR = 0.011, p-value < 0.001), Glyoxylate and dicarboxylate metabolism (FDR = 0.013, p-value < 0.001), Pyrimidine metabolism (FDR = 0.019, p-value < 0.001), and Wnt signaling pathway (FDR = 0.127, p-value = 0.011) were selected in AD. Both were presented in GSEA plots (**Figures 1A, B**). The results implicated that metabolism may be essential in the pathogenesis of psoriasis and AD.

### Immunoreaction Related SsGSEA Analysis

**Figure 2:**
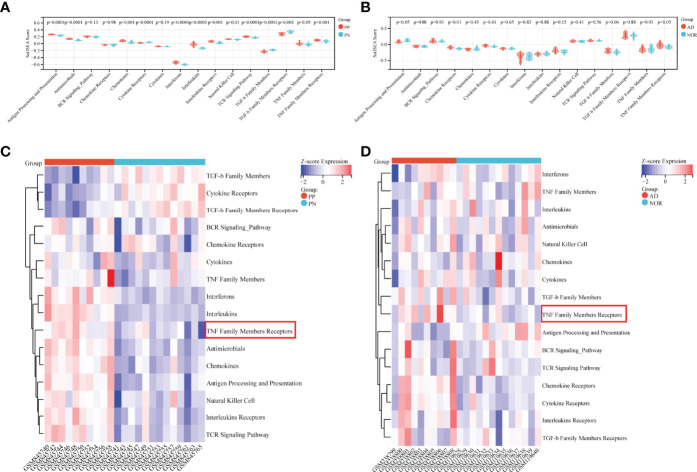
Immunoreaction-related ssGSEA analysis in GSE41664 and GSE5281. **(A, B)** SsGSEA scores of immunoreaction gene lists in each subgroup in GSE41664 and GSE5281. **(C, D)** Heatmap of immunoreaction gene lists in GSE41664 and GSE5281. P-value < 0.05 were considered statistically significant. PN, non-lesion skin tissue of plaque psoriasis; PP, lesion skin tissue of plaque psoriasis; NOR, hippocampus tissue of the normal aged; AD, hippocampus tissue of AD.

SsGSEA Analysis of immunoreaction genes lists conducted in psoriasis and AD could provide specific immune response types involved in the pathogenesis. Based on ssGSEA scores in each sample, all immunoreaction gene lists except three lists (BCR Signaling Pathway, Chemokine Receptors, and Cytokines) obtained enrichment scores that were statistically differential between psoriatic case and control groups (**Figure 2A**). While in AD, only Antigen Processing and Presentation, and three receptors related gene lists: Chemokine Receptors, Cytokine Receptors, TNF Family Members Receptors whose enrichment scores differed between subgroups (**Figure 2B**). Combining heatmaps of two diseases (**Figures 2C, D**), ssGSEA scores of psoriasis were generally higher than AD. While compared with the control group, TNF Family Members Receptors gene lists had higher enrichment scores in the case group either in psoriasis or AD cohort with statistical significance. Interestingly, the Chemokine gene list was enriched in psoriasis cases, while its Receptors gene list was enriched in AD. The results supported the theory that psoriasis is an immune-related disease and suggested that AD was more likely to be affected by hyperinflammatory states as the enrichment scores of inflammatory cytokine receptors were higher in AD than in the normal aged group.

### Identification of DEGs

**Figure 3:**
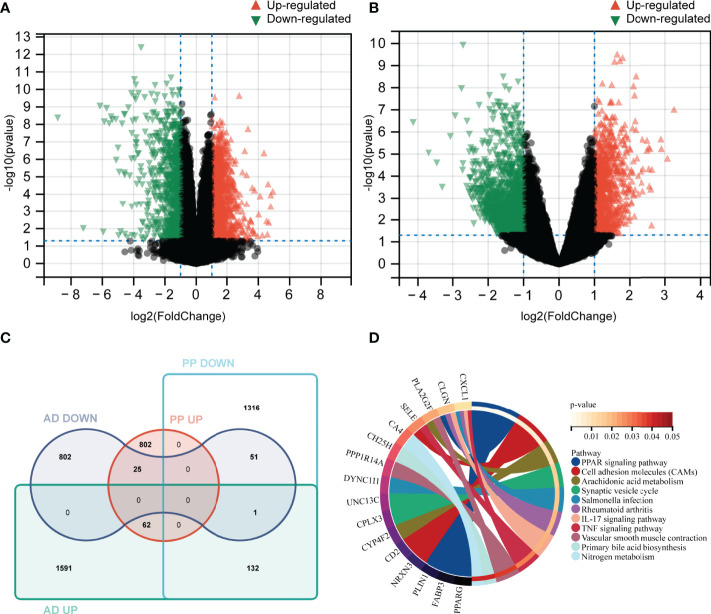
Identification of DEGs and common DEGs, KEGG pathways analysis. **(A, B)** Volcano plot of differentially expressed genes between case and control groups in GSE41664 and GSE5281 (|log2FC| > 1 and p-value < 0.05). **(C)** identification of common DEGs in psoriasis and AD using Venn tools. **(D)** Enriched signature KEGG pathways of common DEGs (p-value <0.05). PP UP, upregulated gene set in lesion skin of plaque psoriasis; PP DOWN, down-regulated gene set in lesion skin of plaque psoriasis; AD UP, upregulated gene set in AD; AD DOWN, down-regulated gene set in AD.

A total of 2389 DEGs (889 upregulated and 1500 downregulated genes) were selected in psoriasis (GSE41664). Meanwhile, 2665 DEGs (1786 upregulated and 879 downregulated genes) were identified in AD (GSE5281). The upregulated DEGs were marked red and those downregulated green in volcano plots (**Figures 3A, B**). These DEGs may be related to the pathogenic processes or clinical status. Therefore, identifying the common DEGs could provide valuable insight into the potential link between two diseases.

### Common Signature KEGG Pathways

Using Venn tools, 113 DEGs (62 upregulated and 51 downregulated genes) were identified as common DEGs in both diseases (**Figure 3C**). In order to explore potential biological pathways in psoriasis and AD, we applied common DEGs to conduct the KEGG pathways analysis. The PPAR signaling pathway, Arachidonic acid metabolism, IL-17 signaling pathway, TNF signaling pathway, Nitrogen metabolism, and other pathways were enriched (**Figure 3D**). Based on these findings, inflammatory and metabolic pathways may be involved in the relationship between AD and psoriasis.

### STFs: PPARG, ZFPM2, ZNF415, HLX and ANHX

**Figure 4:**
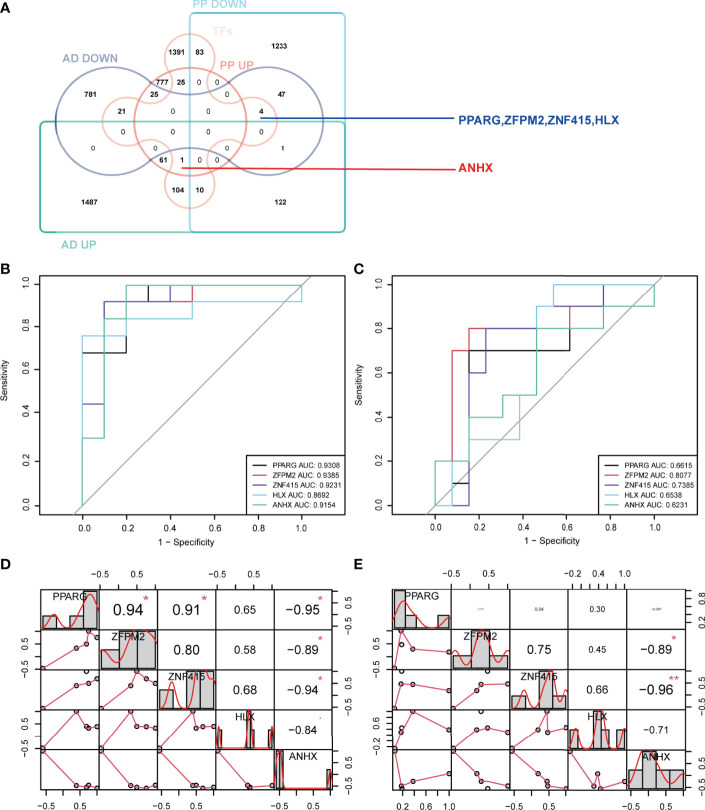
Identification, ROC curve analysis, and correlation analysis of STFs in exploration cohort. **(A)** identification of STFs by Venn diagrams. **(B, C)** ROC curve analysis of each STFs in GSE41664 and GSE5281. STF whose AUC values > 0.60 in psoriasis and AD was considered with diagnostic value; STF whose AUC values > 0.80 was considered with high diagnostic values. **(D, E)** correlation analysis among STFs in GSE41664 and GSE5281. Each cell contains the Pearson correlation coefficient and p-value. P-value < 0.05 and Pearson correlation coefficient (PCC) > 0. 85 were considered as correlated, while p-value < 0.05 and PCC > 0. 95 strongly correlated. * p-value <0.05; ** p-value <0.01. TFs, transcription factors from the Human Transcription Factors Database.

Transcription factors are proteins that bind to specific sequences of DNA, which guide gene expression and participate in many physio-pathological processes (29). Psoriasis and AD are polygenic diseases whose pathogenesis correlates with transcription factors (10). Hence, identifying the common STFs would be helpful in understanding the possible mechanism linking the two diseases. Using the Venn tools, 5 STFs were brought to light. Among these, four genes, PPARG, ZFPM2, ZNF415, and HLX, were down-regulated and ANHX upregulated in psoriasis and AD (**Figure 4A**). Whether these five STFs are valuable in two diseases should be validated.

### Diagnostic Value of STFs in the Exploration Cohort and the Validation Cohort

**Figure 5:**
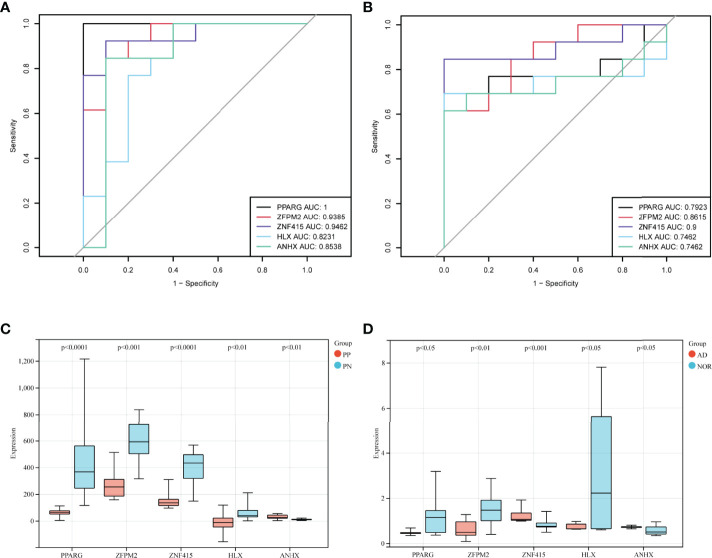
STFs diagnostic values and gene expression level in the validation cohort. **(A, B)** ROC curve analysis of STFs in GSE53552 and GSE48350. **(C, D)** gene expression levels of STFs in GSE53552 and GSE48350. Gene expression levels differentiated (p-value < 0.05) between case and control groups were regarded as statistically significant.

In exploration cohort, the AUC values of STFs (PPARG, ZFPM2, ZNF415, HLX and ANHX) were all > 0.60 in either GSE41664 or GSE5281 (**Figures 4B, C**). The results suggested that all STFs had diagnostic values in psoriasis and AD. We repeated the same analysis in the validation cohort, and the findings turned out to be similar (**Figures 5A, B**). Collectively, all STFs had diagnostic values in psoriasis and AD, especially in psoriasis. Interestingly, ZFPM2 had high diagnostic values for psoriasis and AD either in the exploration cohort or validation cohort (**Table 2**).

**Table 2:** AUC values of STFs in the exploration and the validation cohort of psoriasis and AD.

AUC values STFs	Psoriasis		AD	
	GSE41664 Exploration Cohort	GSE53552 Validation Cohort	GSE5281 Exploration Cohort	GSE48350 Validation Cohort
PPARG	0.9308	1	0.6615	0.7923
ZFPM2	0.9385	0.9385	0.8077	0.8615
ZNF415	0.9231	0.9462	0.7385	0.9
HLX	0.8692	0.8231	0.6538	0.7462
ANHX	0.9154	0.8538	0.6231	0.7462

### STFs Expression Level in the Validation Cohort and the GTEx Portal Database

**Figure 6:**
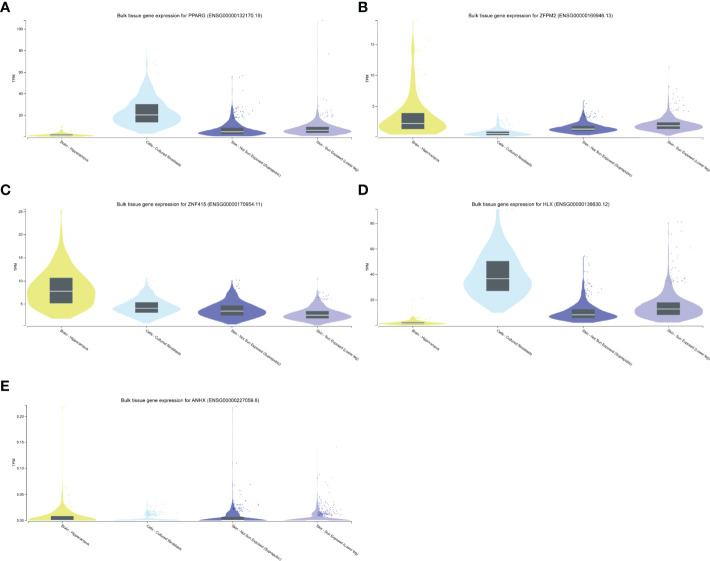
STFs expression levels in GTEx Portal database. **(A–E)** STFs expression levels in skin and hippocampus tissue. Skin tissue contained cultured fibroblasts cells, not sun exposed and sun exposed skin. TPM, Transcripts Per Million.

We performed differentially expressed analysis to explore STFs expression levels in skin and hippocampus tissues. The result suggested that all STFs were differentially expressed between the case and control groups either in psoriasis or AD (**Figures 5C, D**). As was acknowledged in DEGs analysis in the exploration cohort, ZNF415 was downregulated in AD in GSE5281 (**Figure 4A**). However, the expression level of ZNF415 was higher in AD cases than in the normal aged group in GSE48350. This result was contradictory. At the same time, the genotype-tissue expression analysis for STFs illustrated that STFs do express either in normal skin or hippocampus tissue (**Figure 6**). Overall, normal skin and hippocampus tissues express STFs at a basic level, while these two diseases may cause differential expression changes.

### Correlation Analysis of STFs

Binding to DNA, TFs regulate gene expression by recruiting other proteins, like chromatin-modifying enzymes and other TFs (30, 31). By conducting the correlation analysis of STFs, we could investigate whether these STFs have a potential correlation with each other. The results of psoriasis and AD in the exploration cohort suggested that some correlations may exist among STFs. In GSE41664, PPARG was positively correlated with ZFPM2 and ZNF415, whereas adversely with ANHX. However, in GSE5281, PPARG seemed to associate with the other four genes hardly. Meanwhile, ANHX negatively correlated with zinc finger proteins ZNF415 and ZFPM2 in GSE41664 and GSE5281, particularly ZNF415 (**Figures 4D, E**).

### Gene-Gene Interaction Networks

**Figure 7:**
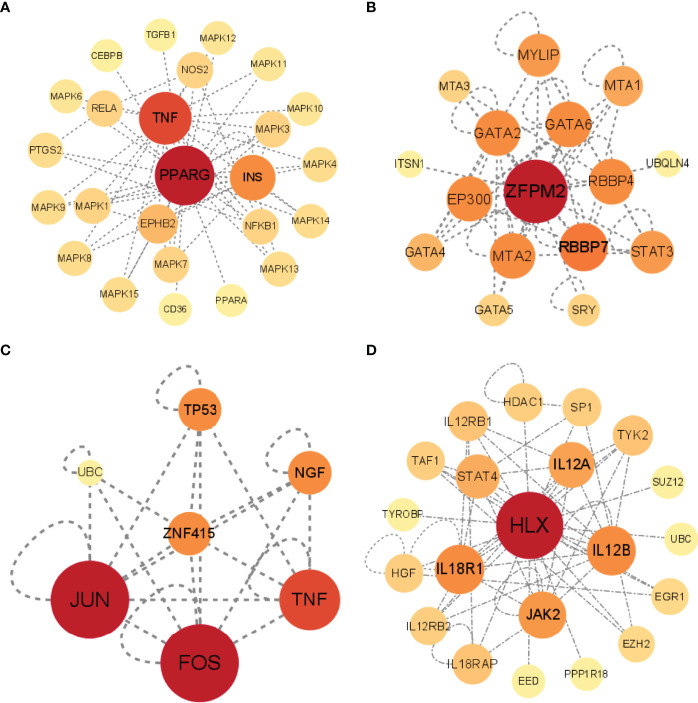
Gene-gene interactions networks of each STF. **(A–D)** Gene networks of 4 STFs. The networks were constructed by the UCSC database, and visualized in circle maps by Cytoscape version 3.9.0.

Besides correlating with STFs themselves, TFs often interact with other genes. The investigation of gene-gene interaction of each STF could provide detailed information on whether these STFs affect or be affected by others. We performed each STF gene network by applying the UCSC database. The results were as follows: TNF, INS, and MAPK families played crucial roles in the PPARG gene network (**Figure 7A**). RBBP7, STAT3, GATA family, especially GATA 2/6, had a close relationship in the ZFPM2 network (**Figure 7B**); JUN, FOX, and TNF in the ZNF415 network (**Figure 7C**); JAK2, TYK2, IL12A, IL12B, STAT4 in HLX network (**Figure 7D**). However, ANHX could not build a gene-gene network in the UCSC database due to a lack of information. The result implicated that TNF, Janus kinase (JAK) family, and Signal transducer and activator of transcription (STAT) family correlated with STFs.

### Chemical-Protein Interaction Networks

**Figure 8:**
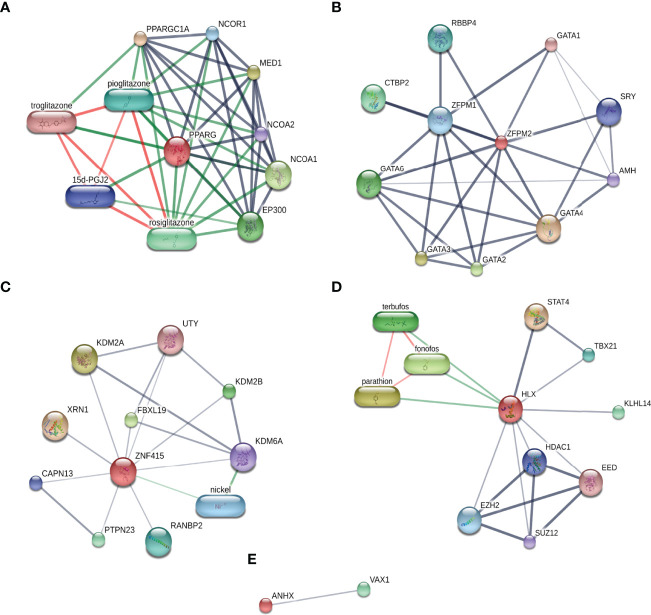
Chemical-protein interaction networks of each STF. **(A–E)** STFs Chemical-protein networks performed in the STITCH database. Stronger associations were represented by thicker lines; protein-protein interactions were shown in grey; chemical-protein interactions in green; and interactions between chemicals in red.

With the aim of investigating the extensive relationship of STFs in gene expression levels, the STITCH database was applied to explore the interactions among STFs, chemicals, and proteins (27). In light of analysis results, PPARG had close relationships with insulin sensitizers, such as pioglitazone, rosiglitazone, and troglitazone, with a combined score of 0.999, 0.999, and 0.998, respectively. (**Figure 8A**). Similar to the gene interaction network, ZFPM2 had close correlations with the GATA family (GATA1, GATA2, GATA3, GATA4, GATA6) ranked scores of 0.869-0.999 (**Figure 8B**). Additionally, it interacted with RBBP4 (0.924), which functions similarly to RBBP7 found in the gene-gene network. HLX was associated with STAT4 (0.933) (**Figure 8D**), the same as the gene-gene network. However, the direct interactions of ZNF415 and ANHX were considered to have no statistical significance, with combined scores of no more than 0.508 (**Figures 8C, E**).

### Protein-Ligand Docking Results

**Figure 9:**
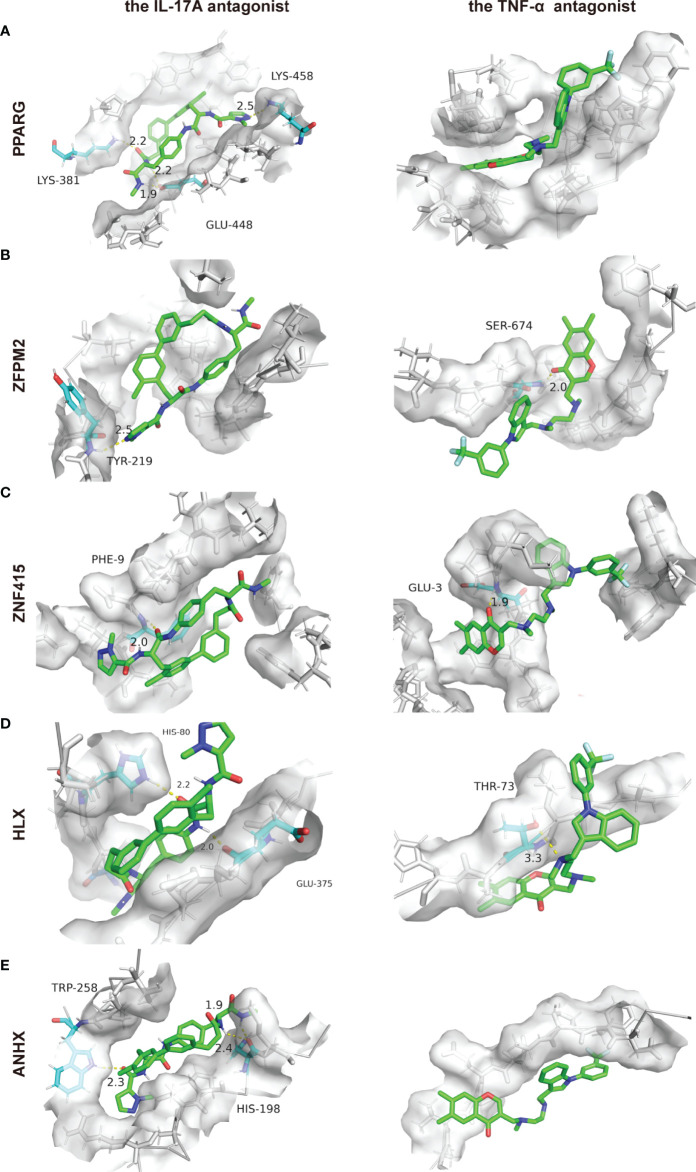
Protein-ligand docking pockets of each STF. **(A–E)** Binding pockets with highest affinities while 5 STFs docking with the IL-17A or the TNF-α antagonist. LYS, Lysine residue; GLU, Glutamate residue; TYR, Tyrosine residue; SER, Serine residue; PHE, Phenylalanine residue; HIS, Histidine residue; THR, Threonine residue; TRP, Tryptophan residue.

By targeting inflammatory cytokines IL-17A or TNF-α, biologics can prevent these inflammatory cytokines from binding to their receptors, thus preventing an inflammatory cascade. In this way, biologics achieve their principal therapeutic effectiveness. As STFs correlated with inflammation-related genes from the above analyses. Besides binding with inflammatory products, we wonder whether they achieved therapeutic benefits through direct combination with STFs in psoriasis and AD. Therefore, we conducted the relative docking analyses. The results illustrated that all STFs had high affinities with IL-17A and TNF-α antagonists. Compared with other STFs, PPARG had the highest affinities when binding with either the IL-17A antagonist or the TNF-α antagonist, followed by ZNF415 and ANHX. Notably, all STFs had higher affinities and more nonpolar chemical bonds when combined with the IL-17 antagonist than the TNF-α antagonist (**Table 3**, **Figure 9**). Collectively, STFs might be the targets of IL-17A and TNF-α antagonists for psoriasis and AD.

**Table 3:** Affinities of binding pockets of STFs and small ligands.

UniProtKB	UniProtKB	Binding energy(kcal/mol)	
		The IL-17A antagonist	The TNF-α antagonist
PPARG	P37231	-7.36	-4.64
ZFPM2	Q8WW38	-4.18	-2.58
ZNF415	Q09FC8	-5.62	-3.16
HLX	Q14774	-4.37	-2.3
ANHX	E9PGG2	-5.56	-2.93

### The Predicted Transcription Factor: ZNF384

**Figure 10:**
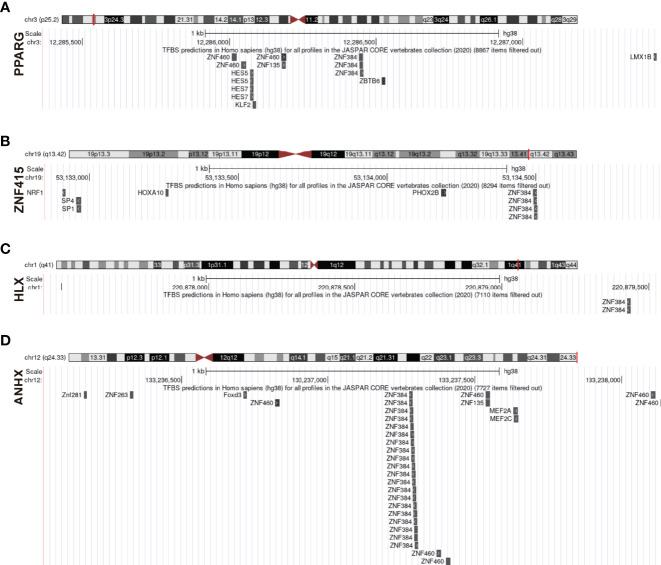
Prediction of upstream transcription factors regulating STFs. **(A–D)** potential upstream transcription factors of STFs. The diagram above presented the gene location in the chromosome; the red line indicated the promoter binding regions (2100bps); the diagram below is a zoomed-in view of the potential promoter regions; all predicted transcription factors that qualified for the threshold were located in their potential binding positions.

We investigated interaction relationships of STFs at different gene expression levels. It was unknown, however, if STFs were controlled by other transcription factors at the gene pre-expression level. Using UCSC genome browser with JASPAR 2020 TFBS track and defining track minimum score as 620 (p-value < 10^-6), ZNF384 was selected as an expected potential transcription factor tuning PPARG, ZNF415, HLX, and ANHX (**Figure 10**). With a threshold of 90% to screen binding profiles in the JASPAR database, ZNF384 was mainly bound to base sequences 908-913 ~ 919-924 upstream of PPARG, mainly to base sequences 1537-1546 ~ 1548-1557 upstream of ZNF415, all to base sequences -2-5 ~ 9-16 upstream of HLX, mainly to base sequences 1141-1166 ~1152-1177 upstream of ANHX. The distinct binding regions predicted in the JASPAR database were in line with approximate binding sites in the UCSC database. In general, ZNF384 might be the potential transcription factor guiding PPARG, ZNF415, HLX, and ANHX expression. Notably, except for ZNF384, ANHX was also potentially regulated by zinc finger proteins ZNF460, ZNF135, and ZNF263.

## Discussion

Psoriasis is an immune-related disease characterized by typical cutaneous manifestations. Recent years have witnessed psoriasis management focusing more on reducing systemic inflammation instead of merely lesion skin improvement (5). AD is a neurodegenerative disease characterized by progressive cognitive decline and memory loss with no effective ways to halt its deterioration. Recent years have witnessed an increased awareness that neuroinflammation drives pathogenesis rather than being a pathophysiological response (32). A nationwide cohort study suggested that psoriasis patients have an increased risk of suffering AD than the general population (8). While using biologics that inhibit inflammatory responses for psoriasis also reduced the risk of developing AD (33). These phenomena implicate that inflammation-related factors may contribute to both diseases. Therefore, we applied bioinformatics methods to explore the potential common molecular mechanism of two diseases.

According to GSEA analysis, biosynthesis and metabolic pathways were enriched in psoriasis and AD. Surprisingly, the pathways enriched in psoriasis may have been associated with amyloid fibrils, the essential component of neuritic plaques of AD. The examples are as follows: functional amyloid fibrils are formed during melanogenesis, just as pathological amyloids are formed in AD (34); ganglio-series of glycosphingolipids mediate the initial aggregation of amyloid β-protein in AD (35); keratan sulfate was found in neuritic plaques, suggesting that keratin sulfate may be crucial to the pathogenesis of neuritic plaques (36). While in immunoreaction-related ssGSEA analysis, the TNF Family Members Receptors gene list was highly enriched in both diseases. The result may illustrate that TNF-α blocking agents protect psoriasis patients from AD on molecular levels (33). Interestingly, the Chemokine gene list was enriched in psoriasis, while its receptor gene list was enriched in AD. Chemokines are pivotal in inflammatory responses for recruiting leukocytes in triggering and aggravating psoriasis (37, 38). As AD was enriched in receptor-related gene lists, we hypothesized that psoriasis increases the risk of developing AD through systemic inflammation products. In KEGG analysis of common DEGs, the IL-17 signaling pathway and TNF signaling pathway were enriched. Meanwhile, some metabolism-related pathways, PPAR signaling pathway, Arachidonic acid metabolism, and Nitrogen metabolism, were also enriched. The analysis implicated that common DEGs functioned in inflammatory and metabolic responses. The IL-17 signaling pathway and TNF signaling pathway are essential in pathological processes of psoriasis. Biologics inhibiting these pathways show great efficacies in treating plaque psoriasis, especially when blocking interleukin-17A (39). Besides being crucial in developing psoriasis, hyperinflammatory responses also play a critical role in AD. Previous studies illustrated that Th17 cells were found in the vicinity of neuritis plaques in AD brains, along with increased pro-inflammatory cytokines (IL-1β, IL-6, CXCL1, and TNF-α) in peripheral blood, which may contribute to the development of Alzheimer’s disease (40). Besides playing essential roles in biochemical homeostasis (41, 42), metabolic pathways contribute to inflammatory responses in several ways. For example, the PPAR signaling pathway involves inflammatory conditions by mediating insulin resistance (41), Arachidonic acid metabolism by synthesizing adipose pro-inflammatory cytokines (43), and Nitrogen metabolism by promoting immune cell proliferation (44). To sum up, our research is in line with previous reports and suggests that chronic inflammation combined with aberrant metabolism is the main risk factor for developing AD in psoriasis.

Polygenic diseases, such as metabolic, immune, and neurological disorders, are characterized by deviant expression of gene sets. Rather than targeting a single gene or its product, reversing accounts of genes through modulation of transcription factors may prove more effective (10). Hence, to further explore the common molecular pathogenesis of psoriasis and AD, we focused our research on transcription factors. We identified 5 STFs (PPARG, ZFPM2, ZNF415, HLX, and ANHX) in common DEGs and investigated their potential roles in psoriasis and AD.

PPARG (Gene Peroxisome Proliferator-Activated Receptor Gamma), a member of the peroxisome proliferator-activated receptor (PPAR) subfamily of nuclear receptors, regulates insulin-stimulated genes expression and involves in the pathology of numerous diseases, including diabetes, atherosclerosis, obesity (45). By targeting PPARG, insulin sensitizers like pioglitazone were developed successfully. Apart from being effective in type II diabetes mellitus, insulin sensitizer pioglitazone alleviates symptoms of plaque psoriasis (46–48). Meanwhile, AD is suggested as a degenerative metabolic disorder attributed to brain insulin resistance and deficits. PPARG agonists act as neuroprotective agents, probably by increasing insulin sensitivity (49). Interestingly, in addition to oral administration, topical delivery of pioglitazone nano-emulsion effectively improve psoriatic skin lesion (50). This phenomenon could hardly be explained by insulin sensitivity improvement. Further exploration suggested its efficacy mainly through inhibiting TH17 differentiation from CD4+ T cells, thus attenuating the IL-17 signaling pathway that leads to psoriasis (50, 51). Meanwhile, pioglitazone could reverse PPARG inhibited status caused by adipose inflammatory cytokines like TNF-α (50–52). Besides being regulated at the post-translational level, PPARG is also modulated by TNF-α at the translational level (53). Hence, except for playing an indispensable role in metabolism, PPARG also closely correlates with inflammatory pathways. Above all, the cross-talk between metabolic disorders and inflammation could explain how PPARG affects psoriasis and AD. Therefore, targeting PPARG and appropriately increasing its expression may serve as a therapeutic target, with great potential to break the positive feedback loop of insulin resistance and hyperinflammatory states.

The transcription factor ZFPM2, also known as FOG Family Member 2(FOG2), is a zinc finger protein that physically interacts with other transcription factors involved in growth and development, including GATA family members GATA-1/2/3/4/5/6 (54, 55). ZFPM2 is mainly expressed in the blood, heart, and nervous system (54). However, there is no evidence that it functions directly in skin tissues. Here, we focus on its functions in immune and metabolic systems, which may commonly impact psoriasis and AD through peripheral blood. Previous clinical trials implicated that hypermethylated ZFPM2 protects against the occurrence and progression of metabolic complications in obesity and could affect immunity-related pathways (56). Meanwhile, some research implicated that decreasing ZFPM2 enhances insulin sensitivity *via* upregulating PPARA expression (57, 58). Given that metabolic deterioration is the risk factor for both diseases, downregulating ZFPM2 should be the protective factor in psoriasis and AD, which contradicts our studies. The possible reasons for this discrepancy are as follows: first, decreased expression of ZFPM2 may serve as the final state biomarker of persistent inflammation in the body rather than one that triggers the inflammatory and metabolic processes (59). This hypothesis may also explain why ZFPM2 got the highest AUC value both in psoriasis and AD. Second, the biological functions of attenuating chronic metabolic inflammation may be suppressed by other factors, like ANHX, which negatively correlated with ZFPM2. Finally, ZFPM2 may possess organ-specific properties that deserve further exploration.

Another aspect that catches our attention is RBBP7 and RBBP4 in gene-gene and chemical-protein interactions. Nucleosome Remodeling and Deacetylase (NuRD) complex subunits RBBP7 and RBBP4 interact with zinc finger-containing proteins (60). They are essential in epigenomic regulation. Dysregulated epigenomes can result in abnormal acetylation or methylation of proteins, which may contribute to many diseases (61, 62). For example, Nikhil Dave et al. reported that downregulation of RBBP7 is positively correlated with neuritic plaque density and pathogenic tau inclusions in AD patients. Interestingly, they further demonstrated that increased RBBP7 was protective against tau-related pathogenesis in animal AD models, consistent with clinical observations (63, 64). Meanwhile, genome-wide studies illustrated that lots of methylated genes differentiate from the lesion and non-lesion skin of psoriatic patients (65). However, there is no direct evidence that RBBP7 or RBBP4 is correlated with psoriasis. Whether ZFPM2 affects the morbidity of psoriasis through epigenomic dysregulation needs further exploration.

ZNF415 is also a member of zinc finger proteins, the largest class of transcription factors in the human genome involved in transcriptional activities and regulation of cellular processes (62). There are five isoforms of ZNF415, namely ZNF415-1 to ZNF415-5, whereas ZNF415-1 plays different roles compared with the other four isoforms. Moreover, the isoforms bind to different DNA-binding sites and regulate various molecular pathways (66). In other words, ZNF415 may play different roles in transcription activities even when its expression levels are the same. Differential splicing of ZNF415 may explain the observation that the expression level of ZNF415 was lower in AD cases in the exploration cohort while higher in the validation cohort.

Previous studies implicated that ZNF415 regulates the transcriptional activities of AP-1 and P53, which is crucial for cell cycle progression and cell death (66). Ap-1 is a dimeric transcription factor mainly containing the Jun (c-Jun, JunB, etc.) and Fos (c-Fos, FosB, etc.) proteins (67). Philipp et al. demonstrated that c-Jun/AP-1 is essential for inducing various pro-inflammatory cytokines in dendritic cells that mediate the onset of psoriatic-like skin inflammation through targeting IL-23 in animal models (68). Compared to c-Jun, JunB seems to play the opposite role. In mice deprived of JunB on the skin, excessive pro-inflammatory factors were produced, subsequently activating the IL-17 signaling pathway, which led to chronic skin inflammation and organ involvement (69). In comparison, upregulation of JunB was proved protective in psoriatic animal models (69, 70). Additionally, clinical studies observed that c-Jun increased while JunB decreased in psoriatic skin compared to the non-lesion region (71, 72). While in AD, stimulating the transcription factor AP-1 enhanced the amyloid-β precursor protein transcription process and eventually exacerbated the neurodegeneration in mice models (73). To summarize, different isoforms of ZNF415 may influence inflammatory pathways by regulating transcriptional activities of different AP-1 components. Additionally, the P53 pathway is essential in immune response, and it regulates STAT3 to induce and maintain inflammatory conditions. Furthermore, p53 also responds to chronic TNF signaling by directly interacting with NF-κB (74, 75). Besides being crucial in inflammation, studies have demonstrated that it influences metabolic pathways through various mechanisms (76). In all, we hypothesize that ZNF415 may be a switch in inflammatory and metabolic pathways. Once it is touched, a series of systemic inflammatory reactions and metabolic disorders may follow.

HLX, namely H2.0-like homeobox, is a Th1-specific transcription factor required for TBX21/T-bet-dependent maturation of Th1 cells. Additionally, HLX is essential in maintaining Th1-specific gene expressions, such as the synthesis of IFN-γ (interferon-gamma) and IL-12RB2 (Interleukin 12 Receptor Subunit Beta 2) (77, 78). Except actively expressed in Th1 cells, HLX-related mRNA expression was even higher in NK cells (77). Instead of increasing IFN-γ secretion in Th1 cell lines, Brian Becknell et al. demonstrated that HLX downregulates IFN-γ expression in NK cells through depletion of STAT4, halting macrophage functions by inhibiting monocyte maturation (79). Importantly, Marc Veldhoen et al. implicated that the absence of HLX could subvert Th1 and Th2 differentiation for the generation of IL-17 producing T cells. Except for regulating Th cells, Zhang et al. identified that HLX deficiency could convert Tr1 cells (type 1 regulatory T cells) to pro-inflammatory cells (80).To sum up, HLX has a variety of roles in different immune cells, but its general function is that low expression correlates with hyperinflammation. Combining the above research, we speculated that downregulating HLX may increase the generation of macrophages and Th17 cells. Thereby, these specific cells excessively produce pro-inflammatory factors like TNF-α, chemokines, and interleukins that are essential to psoriasis and AD.

ANHX, an anomalous homeobox of homo sapiens, is related to DNA binding. According to Pearson correlation analysis, ANHX correlated negatively with the zinc finger proteins ZFPM2 and ZNF415 in both diseases. Additionally, in predicting potential transcription factors analysis, we found that besides ZNF384, other zinc finger proteins ZNF460, ZNF135, and ZNF263 combined with the promoter region of ANHX. Furthermore, the molecular docking analysis implicated that ANHX is highly associated with biologics, especially with the IL-17 antagonist. Here, we speculate that ANHX may cooperate with and be controlled by zinc finger proteins when involved in immune-related or metabolic pathways. Further efforts will be required to confirm the speculation in the future.

As demonstrated by the ssGSEA and KEGG analysis above, the TNF Family Members Receptors gene list, the IL-17 signaling pathway, and TNF signaling pathway were enriched. The analysis suggested that notable immune pathways leading to psoriasis may also exacerbate the onset and progression of AD. Previous studies also reported that biological agents, IL-17A antagonists, and TNF-α antagonists effectively improve cognitive deficits in either AD patients or mice models (81–83). Given that 5 STFs were the selected transcription factors for both diseases, we conducted the associated protein-ligand docking analysis. The results implicated that all STFs had high affinities with IL-17A and TNF-α antagonists. Hence, we speculate that besides combining with corresponding inflammatory cytokines to play their therapeutic roles, biologics may also interact with STFs directly to inhibit the initiation of aberrant inflammatory and metabolic pathways.

In light of predicting potential transcription factor analysis, ZNF384 is the predicted factor that modulates STFs PPARG, ZNF415, HLX, and ANHX. ZNF384 is a zinc finger protein mainly investigated in acute lymphoblastic leukemia (ALL) (84, 85). Studies demonstrated that ZNF384 fusions upregulate the JAK-STAT signaling pathway, thus playing critical roles in ALL (23). The JAK-STAT signaling pathway is critical in immunity responses. It converges many inflammatory pathways, including but not limited to the IL-17 signaling pathway and the TNF signaling pathway. Besides, it also has close relationships with metabolism (86, 87). Since the JAK-STAT signaling pathway was also associated with STFs through network analyses, ZNF384 may tune immune metabolic processes *via* binding to STFs promoter regions.

Psoriasis and AD are both polygenically complex diseases (88, 89). By conducting bioinformatic analysis, PPARG, ZFPM2, ZNF415, HLX, and ANHX were selected as STFs and were further explored the functions in-depth. In summary, we propose an opinion that chronic hyperinflammation and aberrant metabolism increase the risk of developing AD in psoriasis. Our research suggested that besides emphasizing acknowledged comorbidities in psoriasis management, AD deserves attention in psoriasis populations, even in mild to moderate psoriatic states. Using biologics systematically may not only alleviate psoriasis symptoms themselves but reduce the risks of developing AD and other related disorders that are triggered or exacerbated by hyperinflammatory status. Moreover, the predicted STFs upstream transcription factor ZNF384 might be the potential therapeutic target in future drugs development of these two diseases. Our studies give a new perspective on the common mechanisms between psoriasis and AD based on silico experiments. In this study, the expression levels of ZNF415 isoforms need to be further investigated in psoriasis and AD experimentally. Furthermore, the authentic roles of these essential transcription factors need to be further validated *in vitro*. All these will be the focus of our future research.

## Conclusions

Our work proposed that STFs (PPARG, ZFPM2, ZNF415, HLX, and ANHX) mediate the initiation of chronic inflammation and metabolic disorders that increase the risk of developing AD in the psoriasis population. ZNF384 is the potential transcription factor that may modulate STFs PPARG, ZNF415, HLX, and ANHX, thus subsequently triggering hyperinflammatory states and metabolic syndromes. Targeting ZNF384 is a potential therapeutic method for treating plaque psoriasis and AD.

## Data Availability Statement

The datasets presented in this study can be found in online repositories. The names of the repository/repositories and accession number(s) can be found in the article/**Supplementary Material**.

## Author Contributions

SL conceived and guided the research. XY performed data analysis and wrote the manuscript. HS validated the analysis. FL and ZZ revised the manuscript. YC supervised the whole research. All authors contributed to the article and approved the submitted version.

## Conflict of Interest

The authors declare that the research was conducted in the absence of any commercial or financial relationships that could be construed as a potential conflict of interest.

## Publisher’s Note

All claims expressed in this article are solely those of the authors and do not necessarily represent those of their affiliated organizations, or those of the publisher, the editors and the reviewers. Any product that may be evaluated in this article, or claim that may be made by its manufacturer, is not guaranteed or endorsed by the publisher.

## Supplementary Material

Supplementary Table 1 Enriched KEGG pathways in GSEA analysis.
